# Wound‐Healing Efficacy of *Garcinia pedunculata* Fruit Extract: In Vivo and In Silico Validation of Regenerative Mechanisms With Histological Analysis

**DOI:** 10.1002/fsn3.71720

**Published:** 2026-04-02

**Authors:** Kazi Sanjida Tahrim, Md. Mushfiqur Rahman, Md. Sohanur Rahman, Mohammad Abu Sayem, Md. Safayat Hossen Momen, Tanvir Hasan, Md. Jahirul Islam Mamun, Md. Mahmudul Hasan

**Affiliations:** ^1^ Department of Pharmacy International Islamic University Chittagong Chattogram Bangladesh; ^2^ Department of Pharmacy, Faculty of Biological Sciences University of Chittagong Chittagong Bangladesh

**Keywords:** *garcinia pedunculata*, GC–MS analysis, molecular docking, wound healing

## Abstract

*Garcinia pedunculata* Roxb., traditionally used for treating inflammation and infections, remains underexplored for its wound‐healing potential. This study evaluated the wound‐healing efficacy of the *G. pedunculata* fruit ethanolic extract (GP‐EE) using in vivo and in silico approaches. Phytochemical profiling of GP‐EE was performed via Gas Chromatography–Mass Spectrometry (GC–MS). GP‐EE was formulated into 5% and 10% ointments and evaluated in excision and burn wound models in Wistar rats, using 0.2% nitrofurazone as a standard. Wound contraction, epithelialization period, and histopathological features were assessed. Molecular docking was used to examine the interactions of identified bioactives with wound‐healing targets TGF‐β receptor type 1 and IL‐1β, and ADMET properties were evaluated through in silico analysis. GC–MS identified eight major compounds, including mandelic acid (15.05%), gallic acid (7.58%), ellagic acid (4.36%), quercetin (3.45%), and ascorbic acid (4.71%). The 10% GP‐EE ointment showed complete wound closure by day 17 in excision and by day 21 in burn models, significantly faster than the control (*p* < 0.001). Epithelialization time significantly decreased from 20.5 ± 0.8 to 17.33 ± 0.42 days in excision and from 20.24 ± 1.76 to 16.5 ± 0.66 days in burn wounds after 10% GP‐EE treatment (*p* < 0.001). Histology revealed increased fibroblast proliferation, dense collagen deposition, and moderate neovascularization. Molecular docking showed that quercetin and ellagic acid exhibited strong binding affinities for TGF‐βR1 (−9.3 kcal/mol) and IL‐1β (−6.3 kcal/mol), comparable to mefenamic acid. The synergistic antioxidant, anti‐inflammatory, and collagen‐promoting activities of GP‐EE accelerate wound contraction and tissue regeneration. These findings validate the traditional use of *G. pedunculata* and suggest that GP‐EE is a promising natural candidate for the development of phytotherapeutic wound‐healing formulations.

## Graphical abstract

This study demonstrates that *G. pedunculata* fruit possesses potent wound‐healing activity, evidenced by accelerated wound contraction, reduced epithelialization time, enhanced fibroblast proliferation, increased collagen deposition, and improved neovascularization in both excision and burn models.

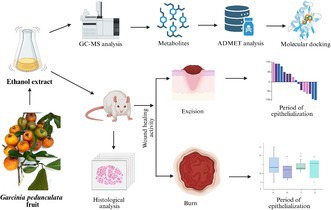

## 1 Introduction

A wound is defined as a disruption or loss of the integrity and function of living tissue, categorized as either acute or chronic (Graves et al. 2022). Wounds are a significant source of disability, healthcare burden, and diminished productivity worldwide. Annually, between 1.2% of the population in industrialized nations experiences chronic wounds (Falanga et al. 2022). Conversely, injuries like burns cause the deaths of 180,000 individuals each year, predominantly in low‐ and middle‐income countries (Gerstl et al. 2024). Chronic wounds induce persistent discomfort and considerable health complications; for example, studies in Northern Europe revealed that foot ulcers are responsible for up to 85% of amputations (Gerstl et al. 2024). In sub‐Saharan Africa and South Asia, 1%–2% of the population encounters chronic wounds at least once in their lifetime (Toppino et al. 2022). In the United States, each year, hundreds of thousands of individuals sustain burn injuries requiring medical attention, while millions live with chronic, non‐healing wounds. Chronic wounds affect about 10.5 million Americans and cost Medicare $22.5 billion annually, with care shifting from outpatient settings to physician offices. Globally, wound care spending reached $148.65 billion in 2022 (Tahouni et al. 2023). In the U.S., approximately 600,000 people sustain burn injuries each year that require emergent care, with an incidence rate of about 257 per 100,000 (Hur et al. 2024; Ivanko et al. 2024). The healing process is a multifaceted biological sequence comprising four primary stages: hemostasis, inflammation, proliferation, and remodeling. The process commences with the development of a blood clot to halt hemorrhaging (Hunt et al. 2023). Galectins provide promising therapeutic targets in hemostasis, inflammation, proliferation, and the maturation and remodeling processes associated with wound healing. Subsequently, the inflammatory phase commences, when immune cells such as neutrophils and monocytes eliminate pathogens and damaged tissue through the secretion of enzymes and reactive agents (Hunt et al. 2023). Wound healing is a complex biological process that restores the structural and functional integrity of damaged dermal tissue. As healing advances, macrophages infiltrate the area, indicating a transition to the later stage of inflammation and promoting tissue repair. The function of macrophages in inflammation and tissue regeneration. The subsequent proliferative phase entails fibroblasts facilitating the formation of new tissue and the generation of blood vessels to sustain it (Peña and Martin 2024). During the final remodeling phase, collagen is reorganized and reinforced to restore the tissue's architecture and resilience. Any disturbance, particularly during the inflammatory phase, may impede healing or result in problems such as persistent wounds (Hasan, Azme, Ekram, et al. 2025; Hasan, Alim, Momen, et al. 2025; Hasan, Azme, Alam, et al. 2025; Hasan, Momen, Alim, et al. 2025).

Traditional medicinal herbs serve as a substantial source of bioactive compounds that exhibit significant anti‐inflammatory and wound‐healing effects (Hasan, Azme, Ekram, et al. 2025; Hasan, Alim, Momen, et al. 2025; Hasan, Azme, Alam, et al. 2025; Hasan, Momen, Alim, et al. 2025). Plant‐derived compounds, such as curcumin, exhibit significant anti‐inflammatory properties through the inhibition of the NF‐κβ and COX‐2 pathways (Ashrafizadeh et al. 2020). Quercetin modulates cytokine production and oxidative stress, demonstrating efficacy as an anti‐inflammatory agent (Boots et al. 2011). Natural compounds such as curcumin and resveratrol have shown the ability to alter TGF‐β signaling, consequently enhancing fibroblast proliferation and extracellular matrix deposition in the context of wound healing (Ashrafizadeh et al. 2020). Polyphenols, including quercetin and epigallocatechin gallate, inhibit IL‐6–mediated inflammation, thereby facilitating tissue repair and regeneration (Zawani and Fauzi 2021). A significant portion of the global population, especially in developing areas, relies on these plants as their primary source of medicine, particularly for addressing inflammation, wounds, burns, and skin disorders (Azme et al. 2025). Herbal medicines are commonly utilized in rural regions due to their accessibility, affordability, and ability to maintain a moist wound environment, which is crucial for effective healing (Hasan, Azme, Ekram, et al. 2025; Hasan, Alim, Momen, et al. 2025; Hasan, Azme, Alam, et al. 2025; Hasan, Momen, Alim, et al. 2025). With the rise of antibiotic resistance and escalating costs of conventional treatments, contemporary medical research is increasingly investigating phytotherapeutic alternatives (Hasan, Azme, Ekram, et al. 2025; Hasan, Alim, Momen, et al. 2025; Hasan, Azme, Alam, et al. 2025; Hasan, Momen, Alim, et al. 2025). Natural compounds affect essential biological processes related to wound healing, such as regulation of inflammation, inhibition of microbial activity, tissue regeneration, and reduction of oxidative stress. This has led to a global focus on acknowledging and validating traditional medicines, seeing them as both cultural heritage and potential sources of innovative, cost‐effective, and scientifically validated therapeutic agents (Schilrreff and Alexiev 2022).

*Garcinia pedunculata* Roxb. (family Clusiaceae) is an underutilized fruit‐bearing species indigenous to Northeastern India and has historically been used to treat digestive disorders, inflammation, and infections (Bhattacharjee and Devi 2021). Recent phytochemical studies have demonstrated that its fruit is an abundant source of phenolics, flavonoids, and chromones, which are chiefly accountable for its pharmacological effects (Hazarika et al. 2024). Numerous investigations have shown that *extracts of G. pedunculata* exhibit considerable anti‐inflammatory, antioxidant, and antibacterial effects (Dutta et al. 2024). The plant demonstrates membrane‐stabilizing properties, prevents protein denaturation, and alleviates oxidative stress and edema in experimental animals, thus suggesting a significant ability to regulate inflammation (Bhattacharjee and Devi 2021). Moreover, its antibacterial activity against *Staphylococcus aureus* , *Escherichia coli* , *Pseudomonas* spp., and *Klebsiella* spp. underscores its promise in thwarting wound infections (Dutta et al. 2024). While research on its wound‐healing properties is limited, the synergistic antioxidant, anti‐inflammatory, and antibacterial effects clearly indicate its therapeutic significance in facilitating tissue repair and expediting wound healing.

Despite its established traditional applications and potential pharmaceutical benefits, the anti‐inflammatory and wound‐healing properties of *G. pedunculata* remain underexplored. This study aims to assess the plant's capacity to regulate inflammation and facilitate wound healing in a rodent model. Molecular docking studies were conducted to elucidate the interactions between the principal bioactive compounds of G. pedunculata and molecular targets implicated in inflammatory control and tissue regeneration. This study seeks to empirically validate the therapeutic efficacy of *G. pedunculata* fruit as a natural remedy for managing inflammation and promoting wound healing, using a combination of in vivo and computational methodologies.

## 2 Materials and Methods

### 2.1 Collection and Extraction of Fruits

Mature but unripe fruits of *G. pedunculata* were harvested in June 2025 from a local garden in Barisal, Bangladesh. The specimen was verified by Professor Dr. Shaikh Bokhtear Uddin from the Department of Botany at the University of Chittagong, and a voucher specimen (MMH/DP/2025/06) was submitted for reference. The GP‐EE was prepared by homogenizing dried fruits and macerating them in ethanol (1:5 w/v) (500 g dried fruit coarse powder to 2.5 L ethanol) for 72 h with occasional shaking. The mixture was filtered through Whatman No. 1 filter paper, and the filtrate was concentrated under reduced pressure using a rotary evaporator at 40°C and 150 mbar to obtain the crude extract. The percentage yield of GP‐EE was calculated using the formula:

$$\%\text{of yield}=\frac{\text{Weight of dried extract}\;\left(\mathrm{gm}\right)}{\text{Weight ofstarting plant material}\;\left(\mathrm{gm}\right)}\times 100$$

43.5 g of extract was found. % of yield of GP‐EE was 8.7%.

### 2.2 Chemicals and Reagents

Analytical‐grade ethanol (≥ 99.9%) and Tween‐80 were obtained from Sigma‐Aldrich (St. Louis, MO, USA), whilst Square Pharmaceuticals PLC supplied urea and other reagents. All other chemicals utilized were of analytical grade and procured from the Department of Pharmacy, University of Chittagong.

### 2.3 Ointment Preparation

The ointment base was formulated in accordance with British Pharmacopeia standards, comprising hard paraffin, cetostearyl alcohol, wool fat, and white soft paraffin in standard proportions (Table 1). Ingredients were heated in a water bath based on their melting points and stirred continuously to ensure uniformity. Two medicated formulations were developed by integrating 5% and 10% (w/w) of GP‐EE into the base. A control ointment comprising only the base was prepared similarly. All ointments were preserved in hermetically sealed containers at room temperature (Hasan, Azme, Ekram, et al. 2025; Hasan, Alim, Momen, et al. 2025; Hasan, Azme, Alam, et al. 2025; Hasan, Momen, Alim, et al. 2025).

**TABLE 1:** Formula of the ointment base.

Ingredient	Percentage (%)
White soft paraffin BP	85
Wool fat BP	5
Hard paraffin BP	5
Cetostearyl alcohol BP	5
Total	100

### 2.4 Acute Dermal Toxicity Study

Dermal toxicity and irritation of GP‐EE were evaluated in accordance with OECD Guideline 410 for repeated‐dose dermal toxicity. Ten male Wistar rats (150–180 g) were divided into two groups of five. The test group received 50% GP‐EE applied topically at 2 mg/cm^2^ to a shaved dorsal area covering ~10% of the body surface, while the control group received sterile water. The treated area was covered with gauze and non‐irritant tape for 24 h daily for 14 consecutive days. Animals were observed daily for clinical signs, erythema, and oedema using the Draize scoring system, and body weights were recorded weekly. At study termination, liver, spleen, kidneys, heart, brain, and lungs were collected and weighed to assess systemic toxicity. Core observations, including irritation scores over time, body weight changes, systemic signs, and organ weights, are summarized in Table S1 (OECD 2017) (Palkhade et al. 2025).

### 2.5 Gas Chromatography–Mass Spectrometry (GC–MS) Analysis

Phytochemical profiling of GP‐EE was performed using a Shimadzu TQ 8040 GC–MS system equipped with an Rxi‐5 ms capillary column (30 m × 0.25 mm × 0.25 μm). Helium was used as the carrier gas at a flow rate of 0.6 mL/min. The injector and interface temperatures were set at 280°C. The oven program started at 60°C (held for 2 min), increased to 280°C at 10°C/min, and held for 10 min. Samples were injected in split mode (split ratio 10:1). Highly polar compounds such as gallic acid, ellagic acid, quercetin, and ascorbic acid were derivatized using BSTFA +1% TMCS prior to analysis to improve volatility. Mass spectra were recorded over 40–350 amu and compared with the NIST 08‐S library for compound identification. Quality control was ensured by injecting derivatized standards and blank runs between samples (Hasan, Azme, Ekram, et al. 2025; Hasan, Alim, Momen, et al. 2025; Hasan, Azme, Alam, et al. 2025; Hasan, Momen, Alim, et al. 2025).

### 2.6 Experimental Animals

Wistar rats were obtained from BCSIR, Chittagong; half were male, and the remainder were female. The average range of the rats was 150–180 g. They were housed in standard polypropylene cages, with five rats per cage. Autoclaved rice husk bedding was used. The animal room was maintained at 22°C–25°C, 50%–60% relative humidity, and a 12 h light:12 h dark cycle. Rats had free access to a standard pellet diet and filtered drinking water throughout the study. After a 7‐day acclimatization period, the animals were randomly assigned to experimental groups, with five rats in each group (Heykants and Mahabir 2016). Randomization was performed using random cage allocation to reduce selection bias. Wound area measurements and histopathological evaluations were performed by an investigator blinded to group allocation to minimize observer bias. All procedures were conducted in accordance with institutional ethical guidelines and in compliance with the ARRIVE guidelines for reporting animal research (Schulz 1995).

- Group I: Vehicle control (base ointment)
- Group II: 5% GP‐EE ointment
- Group III: 10% GP‐EE ointment
- Group IV: Positive control (0.2% w/w nitrofurazone ointment)

### 2.7 Wound‐Healing Activity

#### 2.7.1 Excision Wound Model

Rats were anesthetized with ketamine (80 mg/kg) and diazepam (5 mg/kg). A full‐thickness circular wound (300 mm^2^, 2 mm deep) was created on the shaved dorsolateral flank, disinfected with 70% ethanol. The ointment was applied to fully cover the wound area and maintained until complete wound closure (Hasan, Azme, Ekram, et al. 2025; Hasan, Alim, Momen, et al. 2025; Hasan, Azme, Alam, et al. 2025; Hasan, Momen, Alim, et al. 2025).

#### 2.7.2 Wound Contraction

Wound contraction was evaluated at regular intervals (3 days) using a transparent sheet and a millimeter grid to identify the wound margin by a single observer (Hasan, Azme, Ekram, et al. 2025; Hasan, Alim, Momen, et al. 2025; Hasan, Azme, Alam, et al. 2025; Hasan, Momen, Alim, et al. 2025). The percentage of wound contraction was calculated as:

$$\text{Wound Contraction}\;\left(\%\right)=\frac{\left(\text{Initial wound size}-\text{Wound area}\;\mathrm{on}\;\mathrm{day}\;n\right)}{\left(\text{Initial wound size}\right)}\times 100$$

#### 2.7.3 Epithelialization Period

The epithelialization period was measured as the number of days required for complete shedding of the scab, leaving no fresh wound surface (El‐Sherbeni and Negm 2023).

#### 2.7.4 Burn Wound Model

Burn wounds were generated according to established protocols. While under anesthesia, the dorsal region was shaved and disinfected. Molten beeswax (80°C) was then introduced into a 300 mm^2^ metal cylinder placed on the skin for 10–12 s to create a uniform third‐degree burn lesion. A third‐degree burn was confirmed by the pale, leathery appearance of the wound and absence of bleeding, indicating full‐thickness skin damage. Following cooling and removal, topical treatments were administered daily until the wound closure. The ointment was applied to fully cover the wound area and maintained until complete wound closure. Wound contraction and epithelialization were documented every 3 days (Venter et al. 2015). Post‐burn pain was managed with buprenorphine (0.05 mg/kg, subcutaneously, twice daily) for 3 days (Coutens et al. 2020). Humane endpoints included > 20% body weight loss, severe wound infection, or persistent pain unresponsive to analgesia, at which point animals were euthanized in accordance with institutional animal care and use guidelines (Lilova et al. 2024).

#### 2.7.5 Histopathological Evaluation

Upon conclusion of the experiment, the animals were euthanized, and skin samples were procured and preserved in 10% buffered formalin. Tissue slices (5 μm) were stained with hematoxylin and eosin and microscopically analyzed for fibroblast proliferation, collagen deposition, neovascularization, and epithelial regeneration. Histological outcomes, including fibroblast proliferation, collagen deposition, inflammatory cell infiltration, and neovascularization, were scored from 0 (absent) to 3 (high). Five random fields per section were examined at 400× magnification by two blinded observers. Representative images with 100 μm scale bars were captured. Collagen was quantified from Masson's trichrome‐stained sections using ImageJ, and inflammatory cells were optionally assessed by CD68 immunostaining (Hasan, Azme, Ekram, et al. 2025; Hasan, Alim, Momen, et al. 2025; Hasan, Azme, Alam, et al. 2025; Hasan, Momen, Alim, et al. 2025; Priya et al. 2004).

### 2.8 In Silico Analysis

Molecular docking was performed to forecast interactions between principal bioactive constituents identified in GP‐EE and key receptors involved in wound healing and inflammation, including TGF‐β receptor type‐1 (PDB ID: 6B8Y) and Interleukin‐1β (PDB ID: 6Y8M) (Hasan, Azme, Ekram, et al. 2025; Hasan, Alim, Momen, et al. 2025; Hasan, Azme, Alam, et al. 2025; Hasan, Momen, Alim, et al. 2025). Protein structures were obtained from the RCSB Protein Data Bank; water molecules and heteroatoms were removed; and energy minimization was performed using Swiss‐PdbViewer. A semiflexible docking protocol was applied, keeping the protein rigid while allowing ligand flexibility up to 10 rotatable bonds. The grid (25 × 25 × 25 Å) was centered at *X* = 122.4, *Y* = −5.6, *Z* = 22.8 for TGF‐β receptor and *X* = 8.7, *Y* = 15.3, *Z* = −23.2 for IL‐1β, with exhaustiveness set to 8. These targets were selected due to their key roles in wound healing and inflammation. Model validation was performed by redocking co‐crystallized ligands, yielding RMSD values < 2 Å, confirming the reliability of the docking. For each ligand, nine binding poses were generated. The final pose was selected based on the lowest binding energy and visual agreement with the co‐crystallized ligand (Azme et al. 2025). Ligands were sourced from PubChem, converted to 3D structures, and docked using AutoDock Vina in PyRx. Binding affinities and interaction profiles were depicted utilizing BIOVIA Discovery Studio (Ali, Hoque, et al. 2024; Ali, Noushin, et al. 2024).

### 2.9 ADMET and Drug‐Likeness Prediction

The pharmacokinetic and drug‐likeness characteristics of the discovered compounds were assessed using SwissADME and pKCSM. Parameters, including absorption, distribution, metabolism, excretion, and toxicity (ADMET), were evaluated in accordance with Lipinski's rule of five (Azme et al. 2025).

### 2.10 Animal Ethics and Euthanasia

All experimental procedures were approved by the Ethical Review Board of the University of Chittagong (Ethical approval No. AERB‐FBSCU‐20250615‐(1)) on June 15, 2025, before the start of the study. The experiments were conducted in accordance with the Swiss Academy of Sciences guidelines and the 2013 Animal Euthanasia Guidelines. The principles of the 3Rs, Replacement, Reduction, and Refinement, were strictly followed to ensure animal welfare (Vitale and Ricceri 2022).

### 2.11 Statistical Analysis

Data were presented as mean ± SEM and analyzed using GraphPad Prism v5.0 and SPSS v25. Wound contraction data were analyzed using a mixed‐effects model with treatment as a fixed factor and time as a repeated factor, followed by Tukey's post hoc test. Statistical significance was set at *p* < 0.05, *p* < 0.01, and *p* < 0.001, respectively.

## 3 Result

### 3.1 Acute Dermal Toxicity Analysis

In the repeated dose dermal toxicity bioassay of GP‐EE, no signs of toxicity were observed throughout the study period. The rats attempted to remove the patch within the first 30 min after application. However, no erythema or oedema was noted during the 14‐day observation period in either the control or GP‐EE‐treated groups. The maximum recommended limit dose for acute dermal toxicity is 50% GP‐EE. Accordingly, two lower dose levels, 5% (1/10th) and 10% (1/5th) (w/w) of GP‐EE ointment, were selected. No mortality was recorded in any group during the experiment.

### 3.2 Phytochemical Analysis by GC–MS

**FIGURE 1:**
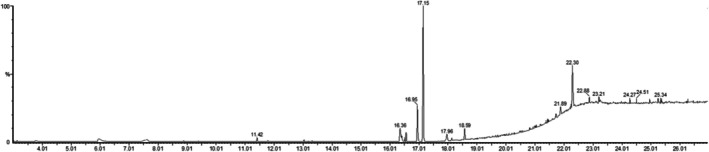
GC–MS chromatogram of GP‐EE. The GC–MS study of GP‐EE identified eight primary phytoconstituents.

**FIGURE 2:**
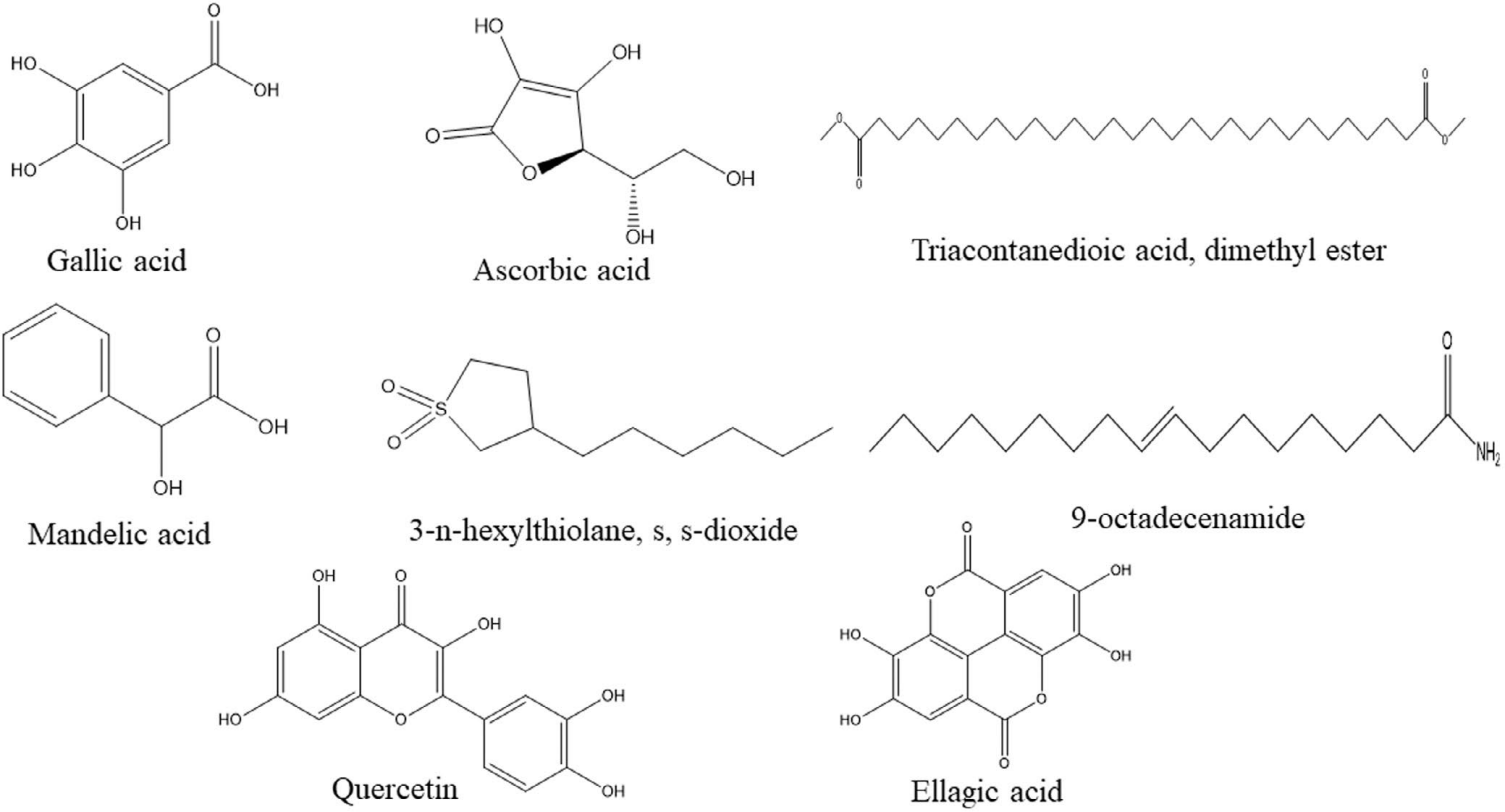
2D Structures of the GC–MS identified phytoconstituents from GP‐EE.

The GC–MS analysis of GP‐EE identified eight main phytoconstituents, with mandelic acid 3,4‐dimethoxy‐, methyl ester (15.05%) as the most abundant component, followed by gallic acid (7.58%), ellagic acid (4.36%), and ascorbic acid (4.71%). Other notable components included 9‐octadecenamide (4.16%), quercetin (3.45%), triacontanedioic acid dimethyl ester (3.02%), and 3‐n‐hexylthiolane, s,s‐dioxide (2.99%) (Figures 1 and 2) & (Table S2).

### 3.3 Analysis of Wound Healing

#### 3.3.1 Excision Wound

##### 3.3.1.1 Measurement of Wound Closure

**FIGURE 3:**
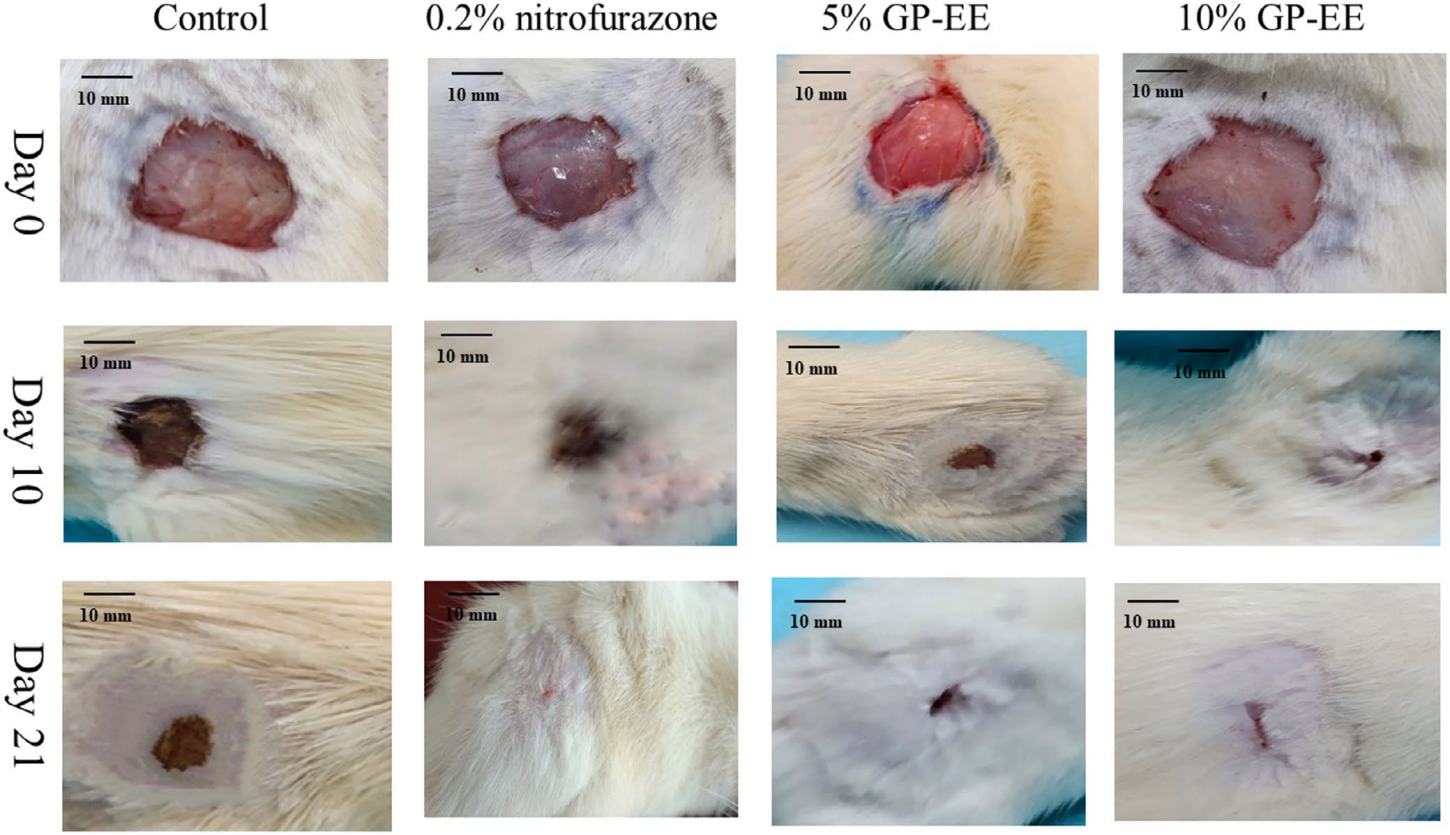
The excision wound healing process as seen visually in rats given GP‐EE. The lesion area improved significantly by day 10 with GP‐EE 10% and by day 17 had healed entirely, consistent with the usual course of ointment therapy.

Compared with vehicle control, the application of 5% and 10% GP‐EE ointments significantly improved wound contraction (*p* < 0.001). The 10% extract group achieved full wound closure (100% contraction) on day 17, results comparable to those of the positive control (0.2% nitrofurazone) (Table 2, Figure 3).

**TABLE 2:** Percentage of wound contraction of rats upon using GP‐EE ointment in excision wounds.

Post‐wounding days	Simple ointment	5% (w/w) GP‐EE ointment	10% (w/w) GP‐EE ointment	0.2% (w/w) nitrofurazone ointment
Day 3	5.14 ± 0.32	7.38 ± 1.65	10.26 ± 0.89	11.00 ± 0.93
Day 6	19.60 ± 2.13	29.16 ± 2.49***	41.00 ± 3.12***	41.79 ± 2.97***
Day 9	43.23 ± 4.12	66.16 ± 3.5***	73.72 ± 3.34***	76 ± 3.12***
Day 12	50.27 ± 3.88	74.44 ± 3.67***	78.97 ± 5.89***	87.11 ± 4.78***
Day 15	71.22 ± 3.12	79.12 ± 4.45***	98.25 ± 0.44***	99.26 ± 0.23***
Day 18	74.15 ± 5.25	83.50 ± 3.66***	100.00 ± 0.10***	100.00 ± 0.01***
Day 21	79.75 ± 4.12	87.50 ± 5.66***	100.00 ± 0.01***	100.00 ± 0.01***

*Note:* The data are expressed as mean ± SEM (*n* = 5) and were analyzed using a mixed‐effects model with treatment as a fixed factor and time as a repeated factor, followed by Tukey's post hoc test. Statistical significance in relation to the control group is denoted as *p < 0.05, **p < 0.01, and ***p < 0.001.

##### 3.3.1.2 Epithelization Period

**FIGURE 4:**
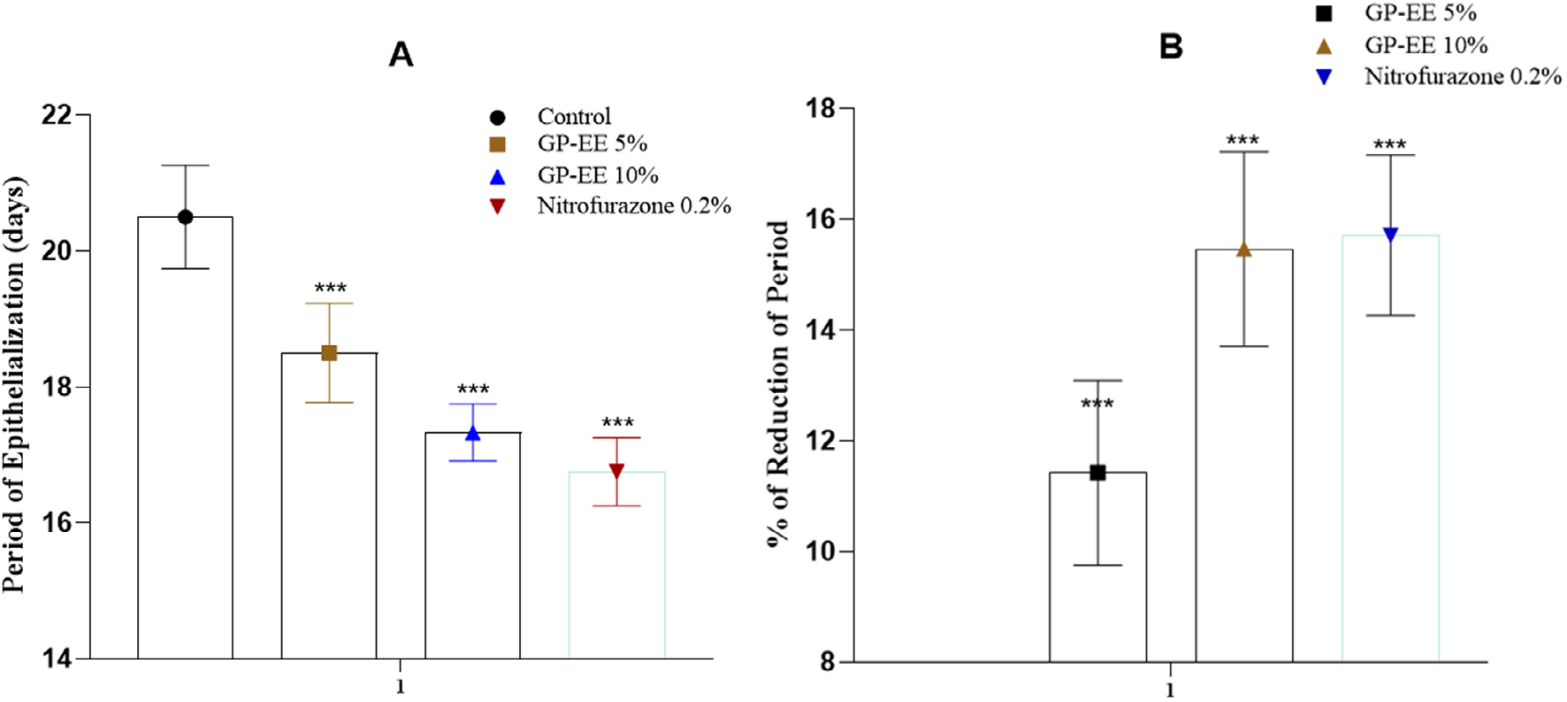
(A) Period of epithelialization in excision wound. (B) The percentage of the reduction period of epithelialization in wound excision. GP‐EE ointment reduced the epithelialization period by a remarkable amount at both doses, comparable to the standard. The data are expressed as mean ± SEM (*n* = 5) and were analyzed using a mixed‐effects model with treatment as a fixed factor and time as a repeated factor, followed by Tukey's post hoc test. Statistical significance in relation to the control group is denoted as *p < 0.05, **p < 0.01, and ***p < 0.001.

The epithelialization duration was significantly reduced in the 10% GP‐EE group (17.33 ± 0.42 days) (*p* < 0.001) compared to the vehicle control (20.5 ± 0.8 days) (*p* < 0.001). In comparison, the percentage of the epithelialization period was reduced to 15.46% ± 1.76% (Figure 4).

##### 3.3.1.3 Histological Analysis

**FIGURE 5:**
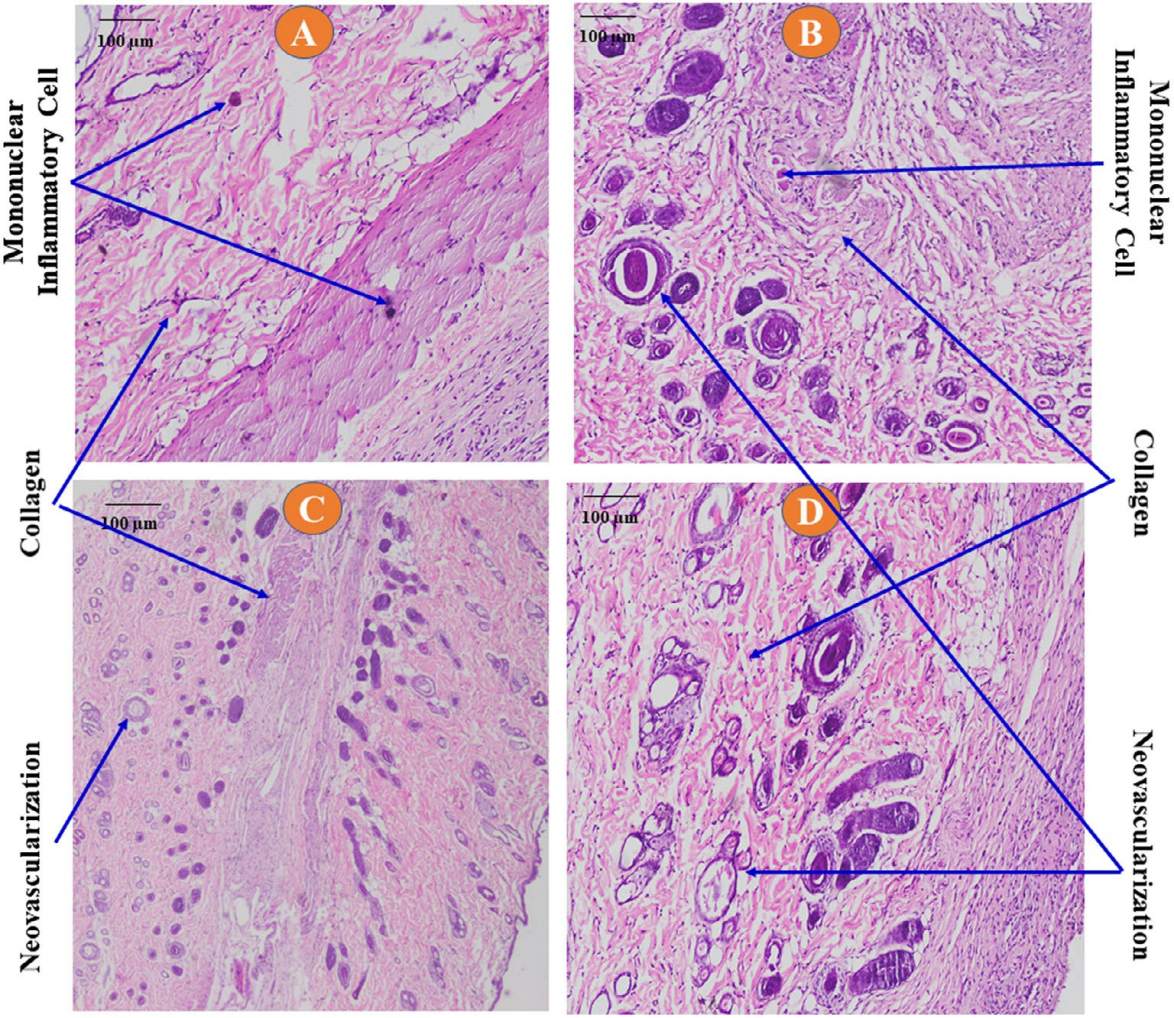
Histological section of the excision wounds. (A) Control; (B) nitrofurazone 0.2%; (C) 5% GP‐EE; (D) 10% GP‐EE. Collagen deposition and neovascularization were present in the healed area, predominantly in the standard and GP‐EE 10% ointment groups.

Comparable to the positive control, the 10% GP‐EE‐treated lesions showed enhanced fibroblast proliferation and marked collagen deposition in histological sections, accompanied by moderate neovascularization (Table 3, Figure 5).

**TABLE 3:** Histological analysis of the excision wound upon treatment.

Group	Fibroblast proliferation	Collagen deposition	Inflammatory cells	Neovascularization
Simple ointment	Low	Low	High	Absent
5% GP‐EE	Low	Moderate	Moderate	Low
10% GP‐EE	High	High	Low	Moderate
0.2% nitrofurazone	High	High	Low	Moderate

#### 3.3.2 Burn Wound

##### 3.3.2.1 Measurement of the Wound Closure

**FIGURE 6:**
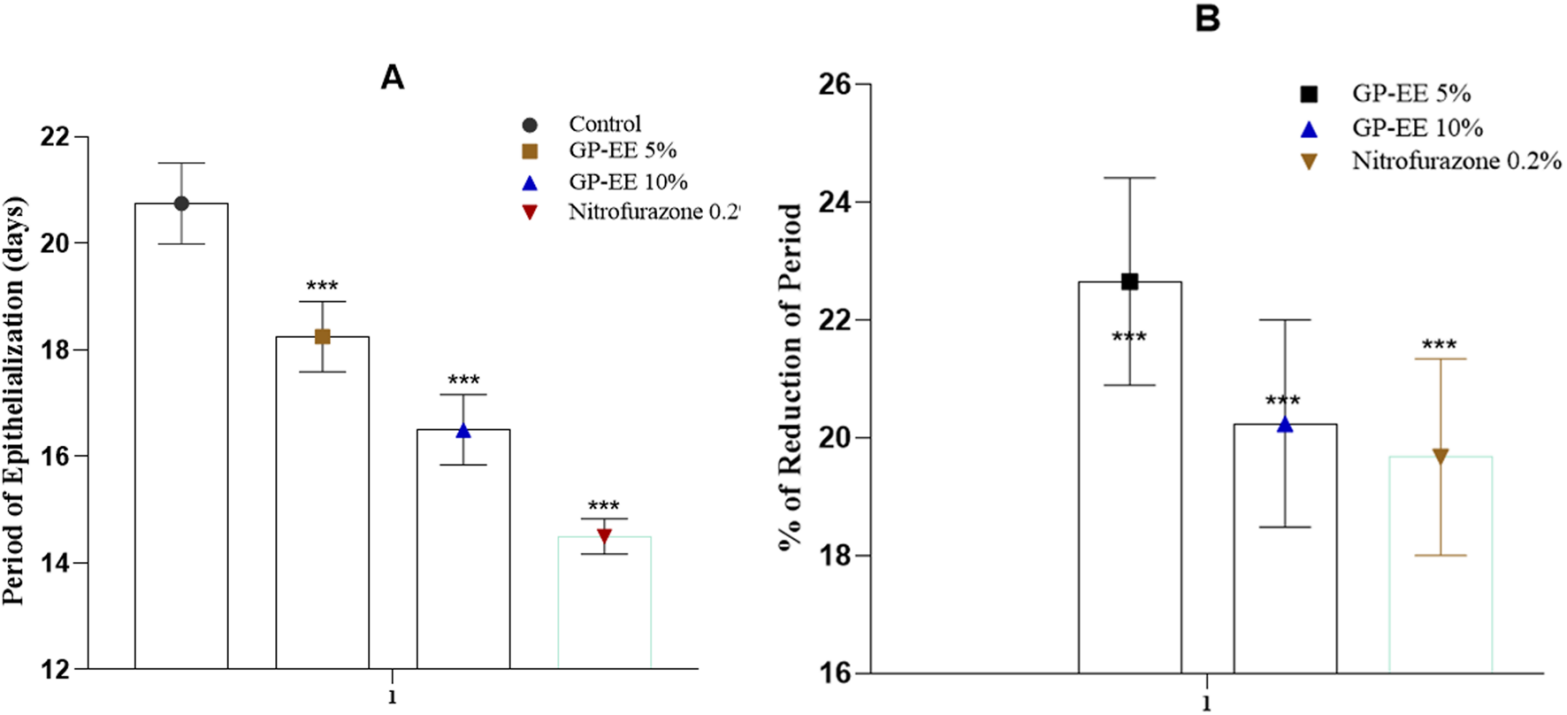
(A) Period of epithelialization in burn excision. (B) The percentage of the reduction period of epithelialization in burn excision. GP‐EE ointment reduced the epithelialization period by a remarkable amount at both doses, comparable to the standard. The data are expressed as mean ± SEM (*n* = 5) and were analyzed using a mixed‐effects model with treatment as a fixed factor and time as a repeated factor, followed by Tukey's post hoc test. Statistical significance in relation to the control group is denoted as *p < 0.05, **p < 0.01, and ***p < 0.001.

**FIGURE 7:**
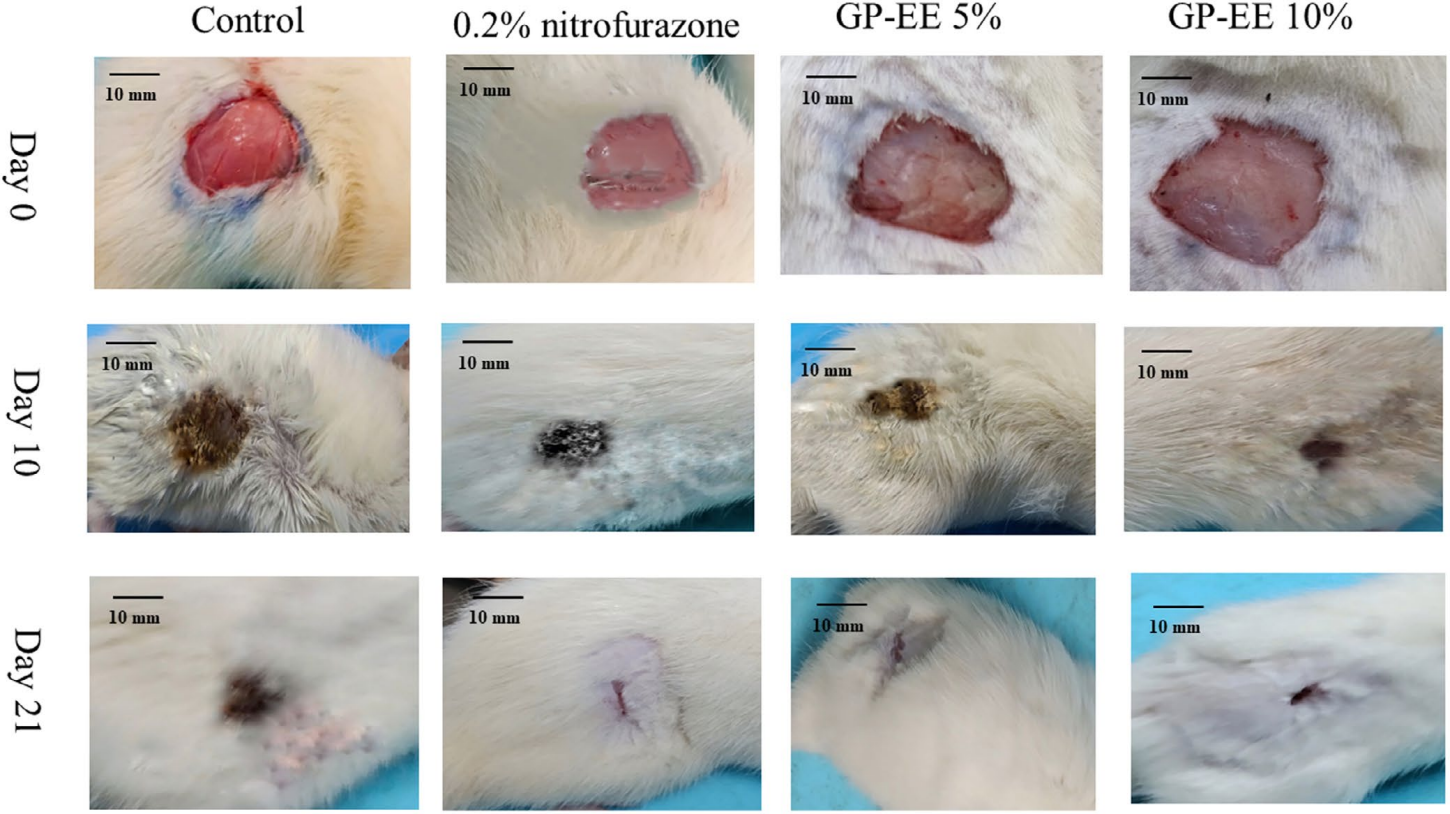
The burn wound healing process as seen visually in rats given GP‐EE. The lesion area improved significantly by day 10 with GP‐EE 10% and by day 18 had closed entirely, consistent with the usual course of ointment therapy.

**FIGURE 8:**
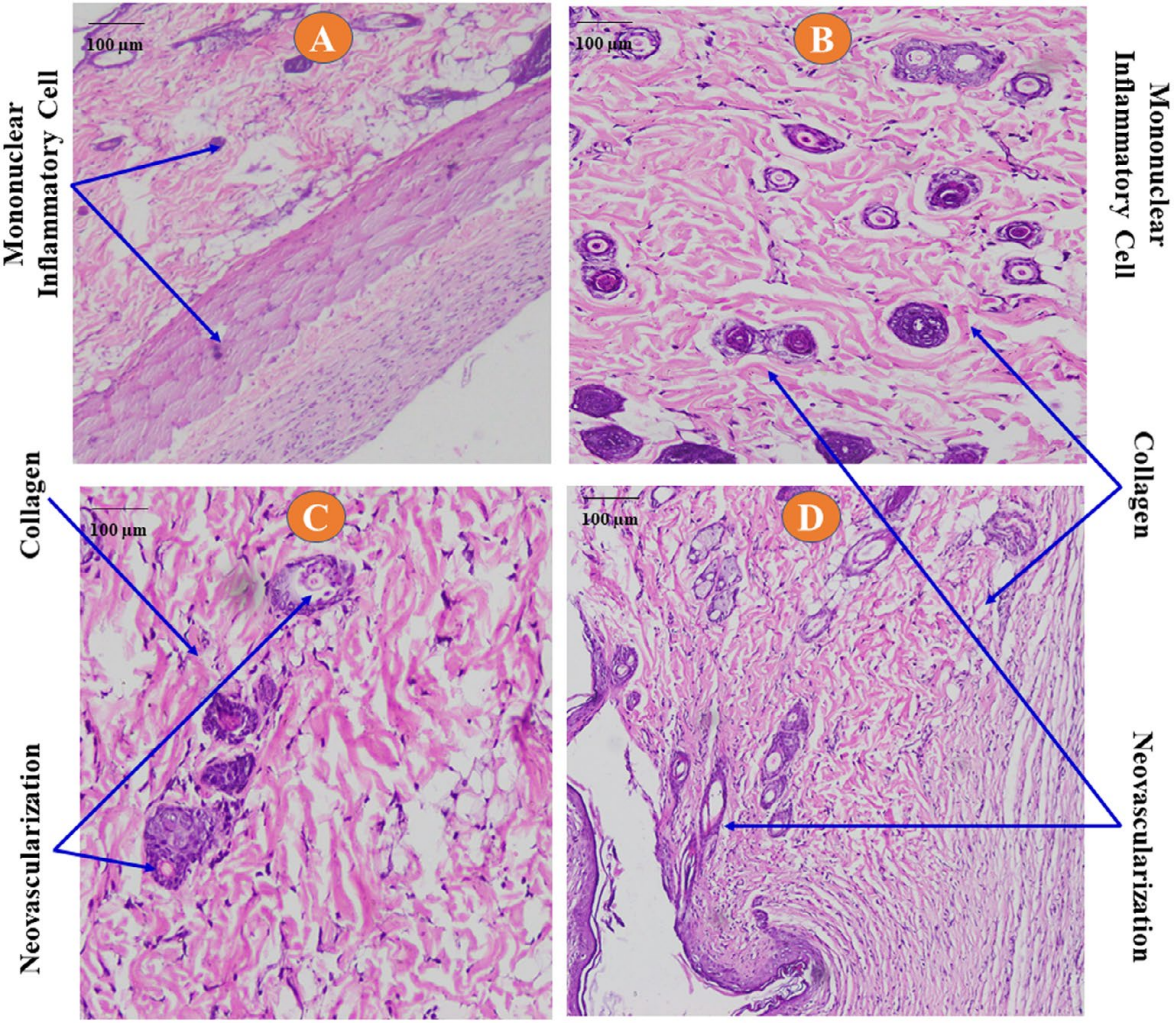
Histological section of the burn wounds. (A) Control; (B) nitrofurazone 0.2%; (C) 5% GP‐EE; (D) 10% GP‐EE. Collagen deposition and neovascularization were present in the healed area, predominantly in the standard and GP‐EE 10% ointment groups.

The 10% GP‐EE ointment from day 9 showed significant wound contraction (*p* < 0.05), and the period of epithelialization decreased to 16.50 ± 0.66 days from 20.75 ± 0.76 days in the control group. Furthermore, there was a 35.58 ± 1.33 decrease in the epithelization period (*p* < 0.05) (Figures 6 and 7) (Table 4). Figure 8 showed neovascularization and collagen deposition.

**TABLE 4:** Percentage of wound contraction of GP‐EE ointment in burn wounds.

Post‐wounding days	Simple ointment	5% (w/w) GP‐EE	10% (w/w) GP‐EE	0.2% (w/w) nitrofurazone ointment
Day 3	3.43 ± 0.55	5.00 ± 0.56	8.91 ± 0.82	9.81 ± 0.33
Day 6	6.35 ± 1.67	19.33 ± 1.76	36.62 ± 0.67	41 ± 2.67***
Day 9	10.73 ± 1.12	33.78 ± 1.67	52.33 ± 1.67*	53.81 ± 2.72***
Day 12	14.54 ± 1.44	57.76 ± 2.44**	66.41 ± 1.40**	77.41 ± 2.88***
Day 15	29.17 ± 2.12	63.04 ± 1.67**	76.67 ± 1.67***	89.16 ± 1.67***
Day 18	33.57 ± 3.12	68.94 ± 1.67**	93.57 ± 1.67***	96.67 ± 2.67***
Day 21	63.57 ± 2.12	72.74 ± 2.25**	100 ± 0.01***	100.00 ± 0.01***

*Note:* The data are expressed as mean ± SEM (*n* = 5) and were analyzed using a mixed‐effects model with treatment as a fixed factor and time as a repeated factor, followed by Tukey's post hoc test. Statistical significance in relation to the control group is denoted as *p < 0.05, **p < 0.01, and ***p < 0.001.

##### 3.3.2.2 Period of Epithelialization

The epithelialization duration was significantly decreased in the 10% GP‐EE group (16.55 ± 0.66 days) (*p* < 0.001) compared to the vehicle control (20.75 ± 0.66 days) (*p* < 0.001). In comparison, the percentage of the epithelialization period was reduced to 20.24% ± 1.76% (Figure 6).

##### 3.3.2.3 Histological Analysis

The 10% GP‐EE‐treated lesions showed substantial collagen deposition and increased fibroblast proliferation in histological sections, along with moderate neovascularization, findings similar to those observed in the positive control (Table 5, Figure 8).

**TABLE 5:** Histological analysis of the burn wound upon treatment.

Group	Fibroblast proliferation	Collagen deposition	Inflammatory cells	Neovascularization
Simple ointment	Low	Low	High	Absent
5% GP‐EE ointment	Low	Moderate	Moderate	Low
10% GP‐EE ointment	High	Moderate	Low	Moderate
0.2% nitrofurazone	High	High	Low	Moderate

### 3.4 Molecular Docking Study

**FIGURE 9:**
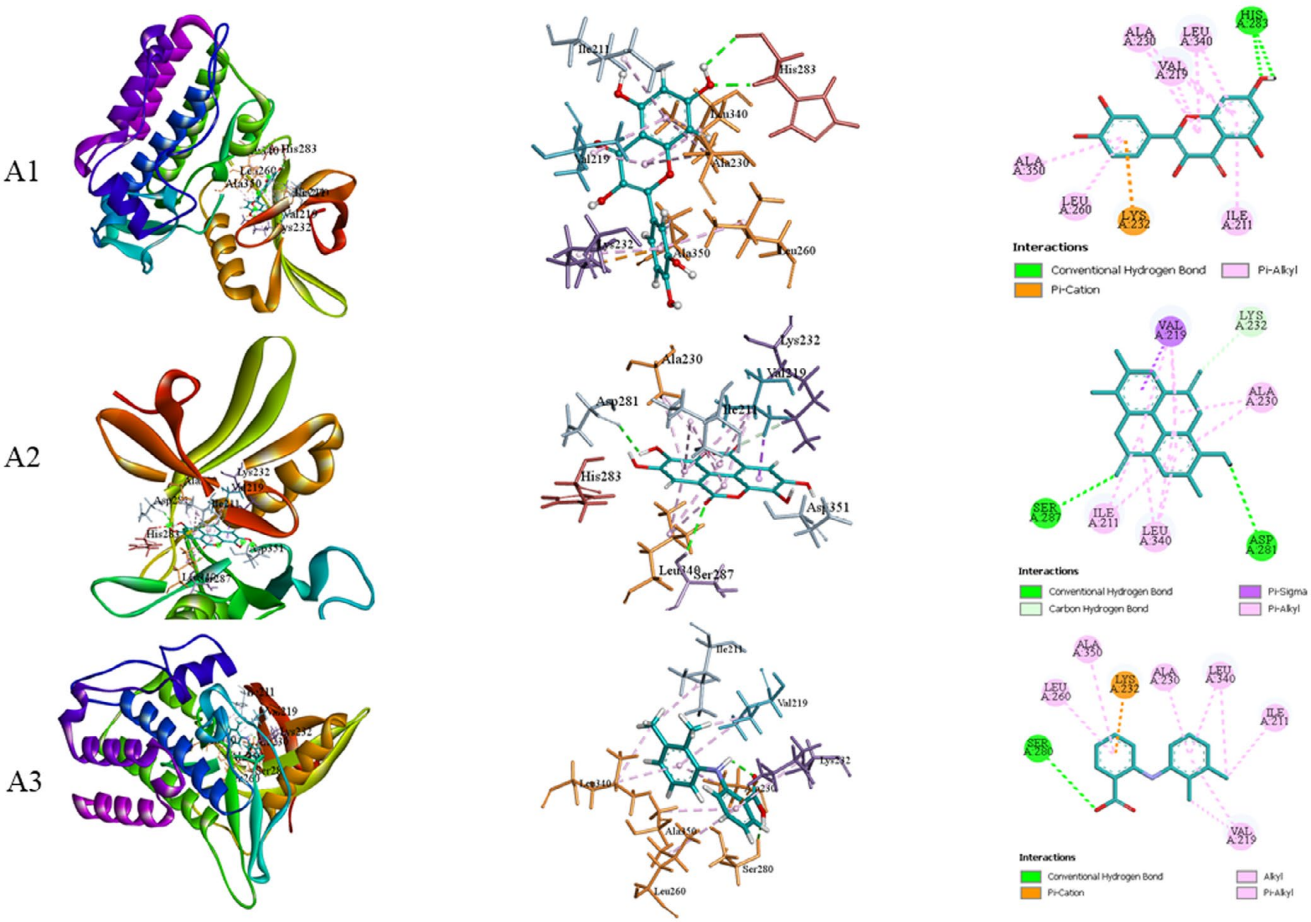
Molecular docking of the bioactive of GP‐EE against TGF‐β receptor type‐1 (PDB ID: 6B8Y) for wound healing properties. A1: Quercetin; A2: Ellagic acid; A3: Mefenamic acid (Standard).

**FIGURE 10:**
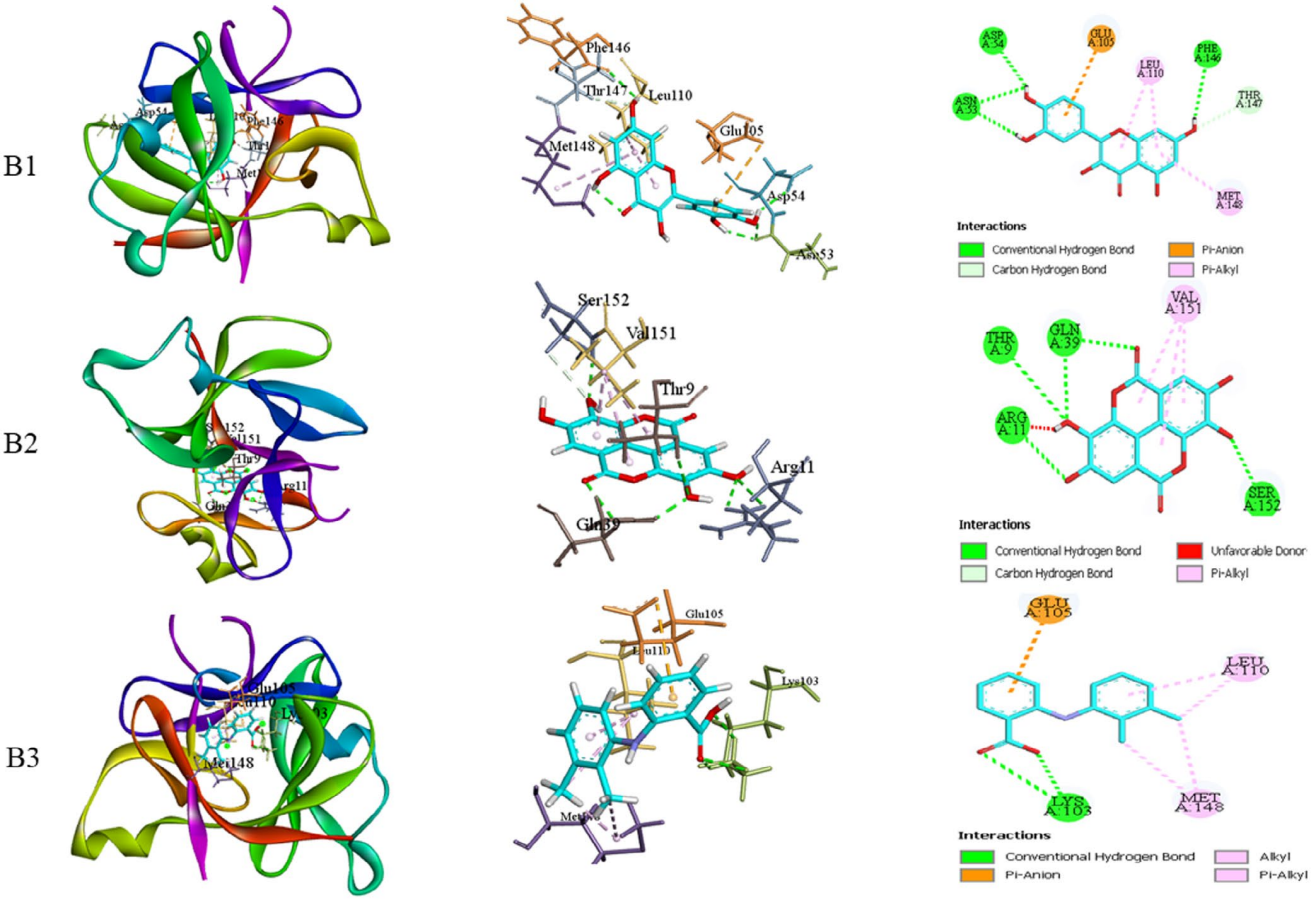
Molecular docking of the bioactive of GP‐EE against Interleukin 1‐β (PDB ID: 6Y8M) for wound healing properties. B1: Quercetin; B2: Ellagic acid; B3: Mefenamic acid (standard).

Metabolites identified in GC–MS analysis were docked with TGF‐β receptor type‐1 (PDB ID: 6B8Y) and Interleukin‐1β (PDB ID: 6Y8M). The binding affinity (Table 6), along with the types of bonds and the amino acids participating in bond formation of the metabolites and standards, is presented in Table 7. Figures 9 and 10 illustrate the leading docked compounds with the specified proteins.

**TABLE 6:** Binding affinity of the metabolites of GP‐EE.

Compound	Binding score	
	6B8Y	6Y8M
Quercetin	−9.3	−6.3
Ellagic acid	−9.1	−5.5
9‐octadecenamide	−6.7	−3.6
Gallic acid	−6.3	−4.2
Mandelic acid, 3,4‐dimethoxy‐, methyl ester	−6.2	−4.5
Triacontanedioic acid, dimethyl ester	−5.7	−3.7
3‐n‐Hexylthiolane	−5.7	−3.9
Ascorbic acid	−5.6	−4.1
Coligand	−11.3	−4.5
Mefenamic acid	−8.8	−5.2

**TABLE 7:** Bonding interactions of the metabolites with proteins.

Protein name	Compound	Binding energy (kCal/mol)	Bond category	Bond type	Amino acid residue
TGF‐β receptor type‐1 (PDB ID: 6B8Y)	Quercetin	−9.3	Hydrogen bond	Conventional hydrogen bond	HIS283, HIS283(2)
			Electrostatic	Pi‐cation	LYS232
			Hydrophobic	Pi‐alkyl	VAL219, ALA230, LEU340, ILE211, VAL219(2), ALA230(2), LEU340(2), LYS232, ALA350, LEU260
	Ellagic acid	−9.1	Hydrogen Bond	Conventional hydrogen bond	SER287, ASP281
				Carbon hydrogen bond	LYS232
			Hydrophobic	Pi‐sigma	VAL219
				Pi‐alkyl	ILE211, VAL219, LEU340, VAL219(2), LEU340(2), ILE211(2), VAL219(3), ALA230, ALA230(2), LEU340(2)
	Mefenamic acid (standard)	−8.8	Hydrogen Bond	Conventional hydrogen bond	SER280
			Electrostatic	Pi‐cation	LYS232
			Hydrophobic	Alkyl	VAL219, ILE211, LEU340
				Pi‐alkyl	LYS232, LEU260, ALA350, VAL219, ALA230, LEU340
Interleukin 1‐β (PDB ID: 6Y8M)	Quercetin	−6.3	Hydrogen Bond	Conventional hydrogen bond	PHE146, ASN53, ASN53 (2), ASP54
				Carbon hydrogen bond	THR147
			Electrostatic	Pi‐anion	GLU105
			Hydrophobic	Pi‐alkyl	LEU110, LEU110 (2), MET148
	Ellagic acid	−5.5	Hydrogen Bond	Conventional hydrogen bond	THR9, ARG11, ARG11(2), GLN39, GLN39(2), SER152
				Carbon hydrogen bond	
			Hydrophobic	Pi‐alkyl	VAL151, VAL151(2), VAL151(3)
	Mefenamic acid (standard)	−5.2	Hydrogen Bond	Conventional hydrogen bond	LYS103, LYS103 (2), LYS103 (3)
			Electrostatic	Pi‐anion	GLU105
			Hydrophobic	Alkyl	MET148, LEU110, MET148 (2)
				Pi‐alkyl	LEU110 (2)

### 3.5 ADMET Analysis

The Absorption, Distribution, Metabolism, Excretion, and Toxicity (ADMET) analysis demonstrated that all GP‐EE compounds were non‐toxic and exhibited good human intestinal absorption with acceptable blood–brain barrier permeability. Most compounds complied with Lipinski's rule of five, except triacontanedioic acid and 9‐octadecenamide. Overall, quercetin, ellagic acid, gallic acid, and ascorbic acid demonstrated favorable drug‐likeness and pharmacokinetic profiles, supporting their therapeutic potential in wound healing (Table 8).

**TABLE 8:** ADMET analysis of the metabolites of GP‐EE.

Compound name	Toxicity parameters		ADME parameters				Lipinski rules				Lipinski's violation ≤ 1
	AMES toxicity	Carcinogens	AOT	HIA	HOB	BBB	MW (g/mol) < 500	HBA < 10	HBD < 5	Log *p* ≤ 5	
Gallic acid	NAT	NC	III	0.8051	0.56	0.6225	170	5	4	0.5	0
Ascorbic acid	NAT	NC	IV	0.6559	0.56	0.8532	176	6	4	−1.41	0
Triacontanedioic acid, dimethyl ester	NAT	NC	III	0.7681	0.17	0.9654	510	4	0	10.26	2
Mandelic acid, 3,4‐dimethoxy‐, methyl es	NAT	NC	III	0.935	0.55	0.7557	226	5	1	0.59	0
3‐n‐hexylthiolane, s, s‐dioxide	NAT	NC	III	0.9927	0.55	0.9769	204	2	0	3.47	0
9‐octadecenamide	NAT	NC	III	1	0.55	0.9972	281	1	1	5.51	1
Quercetin	NAT	NC	II	0.965	0.55	0.5711	302	7	5	1.99	0
Ellagic acid	NAT	NC	II	0.7199	0.55	0.5641	302	8	4	1.31	0

Abbreviations: AOT, acute oral toxicity; BBB, blood–brain barrier; HBA, hydrogen bond acceptors; HBD, hydrogen bond donors; HIA, human intestinal absorption; HOB, human oral bioavailability; Log P, octanol–water partition coefficient; MW, molecular weight; NAT, non‐ames toxic; NC, non‐carcinogenic.

## 4 Discussion

The GC–MS analysis of GP‐EE revealed eight principal phytochemicals, including gallic acid (7.58%), ascorbic acid (4.71%), ellagic acid (4.36%), quercetin (3.45%), and mandelic acid 3,4‐dimethoxy‐, methyl ester, among others, establishing a robust biochemical foundation for its wound‐healing activity (Dunphy et al. 1956; Li et al. 2019; Yan et al. 2025). Gallic and ellagic acids are known to promote fibroblast proliferation, collagen synthesis, and extracellular matrix production‐key processes in the proliferative phase of wound repair (Yan et al. 2025). Quercetin exerts potent anti‐inflammatory and pro‐angiogenic effects by suppressing cytokines such as TNF‐α and IL‐6 while promoting VEGF‐mediated angiogenesis, thereby enhancing granulation tissue formation and tissue oxygenation, as reported by Lin et al. (2025). Dunphy et al. demonstrated that ascorbic acid supports collagen biosynthesis and maturation by serving as a cofactor for prolyl and lysyl hydroxylases, ensuring the stabilization and cross‐linking of collagen fibers and facilitating epithelialization (Dunphy et al. 1956). Collectively, these compounds may act synergistically to mitigate oxidative stress, regulate inflammation, and stimulate fibroblast activity, thereby enhancing wound closure and tissue regeneration.

In vivo evaluation further confirmed the wound‐healing efficacy of GP‐EE in both excision and burn wound models. Topical application of 5% and 10% GP‐EE ointments significantly accelerated wound contraction, with the 10% formulation achieving complete closure by day 17 in excision wounds and by day 21 in burn wounds. Histopathological analyses corroborated these findings, revealing increased fibroblast proliferation, dense collagen deposition, and moderate neovascularization, which collectively support efficient tissue remodeling (El‐Sherbeni and Negm 2023; Priya et al. 2004). These outcomes are consistent with prior findings on related *Garcinia* species, such as *G. mangostana* , *G. kola* , and *G. cambogia*, which exhibit complementary antioxidant, anti‐inflammatory, and regenerative activities—strengthening the pharmacological evidence at the genus level (do Espirito Santo et al. 2020; Noreen et al. 2023; Krishnapriya et al. 2025).

At the molecular level, TGF‐β receptor type‐1 (TGF‐βR1) and interleukin‐1β (IL‐1β) play pivotal roles in orchestrating the wound‐healing process (Bouafia et al. 2025). TGF‐βR1 regulates fibroblast proliferation, collagen production, and extracellular matrix deposition, which are essential for granulation tissue formation and scar remodeling (Ji et al. 2024). In contrast, IL‐1β initiates the inflammatory phase by recruiting immune cells, clearing debris, and promoting the transition from inflammation to proliferation (Lopez‐Castejon and Brough 2011). In molecular docking, the binding site was defined by residues surrounding the co‐crystallized ligand in each structure. Docked ligands were examined for overlap with the co‐crystallized ligand and for hydrogen bonds, hydrophobic interactions, and π–π stacking. The analysis confirmed that the ligands occupied the same binding pocket and interacted with key residues essential for receptor activity (Ali, Hoque, et al. 2024). Molecular docking analysis revealed that quercetin and ellagic acid exhibited the highest binding affinities toward both TGF‐βR1 and IL‐1β, indicating their potential to modulate these targets. Quercetin may enhance fibroblast proliferation and collagen synthesis via TGF‐β activation while suppressing IL‐1β–mediated inflammation, facilitate wound healing (Ji et al. 2024; Lopez‐Castejon and Brough 2011; Lopez‐Castejon and Brough 2011). Similarly, ellagic acid may downregulate IL‐1β and NF‐κβ pathways while promoting angiogenesis and extracellular matrix remodeling through TGF‐β–dependent mechanisms (Bouafia et al. 2025; Ji et al. 2024). However, it requires separate experimental validation to confirm effects on fibroblast proliferation, collagen synthesis, inflammation, and angiogenesis. The dermal toxicity of GP‐EE was assessed in accordance with OECD guidelines. Predicted ADMET profiles suggested good absorption, metabolic stability, and low toxicity, though it was used as a topical formulation. However, these results are based on computational analysis and lack experimental validation. Moreover, the predictions are more relevant to potential systemic use than to the topical wound‐healing model used in this study.

The ethnomedicinal relevance of *G. pedunculata* is well supported by pharmacological and molecular evidence. The combined actions of phytochemicals present in GP‐EE contribute to the extract's ability to promote fibroblast proliferation, collagen deposition, angiogenesis, and tissue remodeling while mitigating oxidative stress and inflammation. The efficacy of 5% and 10% GP‐EE ointments, as evidenced by accelerated wound contraction, shorter epithelialization periods, and improved histological architecture, provides scientific validation for their traditional use in treating cuts, burns, and ulcers. Overall, GP‐EE exhibits significant potential as a natural therapeutic agent by integrating antioxidant, anti‐inflammatory, and tissue‐regenerative mechanisms, offering a promising foundation for developing novel wound‐healing formulations.

## 5 Limitations and Prospects

GC–MS was employed because it is well‐suited to identifying volatile and semi‐volatile constituents and is widely used in preliminary phytochemical screening. However, this approach does not capture non‐volatile or highly polar compounds. Future studies should incorporate LC–MS or HPLC to achieve a more comprehensive phytochemical profile. Wound contraction was measured repeatedly in the same animals. The use of one‐way ANOVA at individual time points does not fully account for within‐subject variability. Future work should employ repeated‐measures or mixed‐effects models to more effectively assess treatment‐by‐time effects. Another limitation is the lack of direct biomarker analysis to confirm the molecular mechanisms underlying the observed wound‐healing effects. Further studies should focus on isolating and characterizing specific bioactive compounds, evaluating their pharmacokinetics and safety, and mechanistically validating their effects using relevant molecular and biochemical biomarkers. In addition, development of optimized formulations, such as gels, creams, or bioactive dressings, may enhance translational potential and practical application in modern wound care.

## 6 Conclusion

This study demonstrates that GP‐EE exhibits considerable wound‐healing capabilities, as evidenced by accelerated wound contraction, reduced epithelialization time, increased fibroblast proliferation and collagen accumulation, and moderate neovascularization in both excision and burn wound models. The therapeutic effects may be robustly substantiated by its phytochemical composition, which includes gallic acid, ellagic acid, quercetin, ascorbic acid, and mandelic acid derivatives. These compounds function synergistically through antioxidant, anti‐inflammatory, and collagen‐enhancing processes. Molecular docking indicates that these chemicals may modulate essential wound‐healing targets, including TGF‐βR1 and IL‐1β, thereby providing a probable mechanistic insight into their bioactivity. These findings provide scientific corroboration of the conventional use of *G. pedunculata* in wound treatment. Looking ahead, GP‐EE shows promise as a potential candidate for developing natural, plant‐based wound‐healing medicines.

## Author Contributions

**Kazi Sanjida Tahrim:** investigation, software, data curation, formal analysis, visualization, writing – original draft, writing – review and editing. **Md. Mushfiqur Rahman:** formal analysis, visualization, writing – original draft, writing – review and editing. **Md. Sohanur Rahman:** formal analysis, visualization, writing – original draft. **Mohammad Abu Sayem:** formal analysis, visualization, writing – original draft. **Md. Safayat Hossen Momen:** formal analysis, visualization, writing – original draft. **Tanvir Hasan:** formal analysis, visualization, writing – original draft. **Md. Jahirul Islam Mamun:** visualization, writing – original draft, writing – review and editing. **Md. Mahmudul Hasan:** conceptualization, investigation, software, data curation, formal analysis, visualization, writing – original draft, writing – review and editing.

## Funding

The authors have nothing to report.

## Ethics Statement

The Ethical Review Board Approval No: AERB‐FBSCU‐20250615‐(1) approved the study, adhering to the Swiss Academy of Sciences guidelines and the 2013 Animal Euthanasia Guidelines.

## Conflicts of Interest

The authors declare no conflicts of interest.

## Supporting information


**Table S1:** Acute toxicity data.
**Table S2:** GC–MS analysis of GP‐EE.

## Data Availability

The data that support the findings of this study are available on request from the corresponding author. The data are not publicly available due to privacy or ethical restrictions.
