# Supplementary material for: Wound‐Healing Efficacy of Garcinia pedunculata Fruit Extract: In Vivo and In Silico Validation of Regenerative Mechanisms With Histological Analysis
Source: Food Sci Nutr. 2026 Apr 2;14(4):e71720. doi: 10.1002/fsn3.71720 (PMC13052341; doi:10.1002/fsn3.71720)
Supplement: Supplementary file 1 — Table S1: Acute toxicity data. Table S2: GC–MS analysis of GP‐EE. [file FSN3-14-e71720-s001.docx]

**Table S1:** Acute toxicity data

| **Parameter** | **Control (n=5)** | **GP-EE 50% (n=5)** |
| --- | --- | --- |
| **Dose (topical)** | 0 mg/cm² | 2 mg/cm² |
| **Erythema score (Draize, 0–4)** | 0 throughout | 0–1 (transient, days 1–2), 0 thereafter |
| **Oedema score (Draize, 0–4)** | 0 throughout | 0 throughout |
| **Body weight (g)** | Day 0: 162 ± 5  Day 7: 168 ± 6  Day 14: 174 ± 7 | Day 0: 164 ± 6  Day 7: 170 ± 5  Day 14: 176 ± 6 |
| **Systemic signs** | None | None |
| **Organ weights (g, mean ± SEM)** |  |  |
| Liver | 7.8 ± 0.5 | 8.0 ± 0.4 |
| Kidneys | 1.6 ± 0.1 | 1.7 ± 0.1 |
| Spleen | 0.7 ± 0.05 | 0.7 ± 0.06 |
| Heart | 1.2 ± 0.1 | 1.2 ± 0.1 |
| Brain | 2.0 ± 0.1 | 2.0 ± 0.1 |
| Lungs | 1.5 ± 0.1 | 1.5 ± 0.1 |

**Table S2:** GC-MS analysis of GP-EE

| **SL** | **Compound Name** | **M.W.** | **Formula** | **% of Area** | **Retention Time (min)** | **Retention Index (RI)** | **Library Match Score (%)** |
| --- | --- | --- | --- | --- | --- | --- | --- |
| 1 | Gallic acid | 170 | C₇H₆O₅ | 7.58 | 11.42 | 1165 | 94 |
| 2 | Ascorbic acid | 176 | C₆H₈O₆ | 4.71 | 16.95 | 1048 | 92 |
| 3 | Triacontanedioic acid, dimethyl ester | 510 | C₃₂H₆₂O₄ | 3.02 | 18.69 | 3205 | 90 |
| 4 | Mandelic acid | 226 | C₁₁H₁₄O₅ | 15.05 | 21.89 | 1890 | 95 |
| 5 | 3-n-hexylthiolane, S,S-dioxide | 204 | C₁₀H₂₀O₂S | 2.99 | 22.30 | 2065 | 91 |
| 6 | 9-Octadecenamide | 281 | C₁₈H₃₅ON | 4.16 | 24.27 | 2548 | 93 |
| 7 | Quercetin | 302 | C₁₅H₁₀O₇ | 3.45 | 24.51 | 2685 | 92 |
| 8 | Ellagic acid | 302 | C₁₄H₆O₈ | 4.36 | 24.34 | 3468 | 94 |
